# The correlation between on-farm biosecurity and animal welfare indices in large-scale turkey production

**DOI:** 10.1016/j.psj.2024.104598

**Published:** 2024-11-27

**Authors:** László Kovács, Christopher René Klaucke, Máté Farkas, Mikolt Bakony, Viktor Jurkovich, László Könyves

**Affiliations:** a Department of Animal Hygiene, Herd Health and Mobile Clinic, University of Veterinary Medicine, 1078 Budapest, Hungary; b Department of Digital Food Science, University of Veterinary Medicine, 1078 Budapest, Hungary; c Centre for Translational Medicine, Semmelweis University, 1085 Budapest, Hungary; d Centre for Animal Welfare, University of Veterinary Medicine, 1078 Budapest, Hungary

**Keywords:** Biosecurity, Animal welfare, Turkey flocks

## Abstract

This research aimed to determine the association between biosecurity and animal welfare measures in large-scale fattening turkey farms in Hungary. Large-scale farms raising male fattening turkeys across Hungary were contacted, with 24 agreeing to participate. The Biocheck.UGent questionnaire was used to evaluate biosecurity measures, and Animal Welfare Indicators (AWIN) protocol was used for welfare assessment. The association between biosecurity and welfare indicators was investigated by correlation testing and prediction accuracy using random forest classification. The areas of biosecurity that were shown to be closely linked to the welfare index were the organization of the farm (farm management, supply of materials), the control and hygienic measures implemented between farm structures, cleaning and disinfection measures on the farm, and control and hygienic measures implemented in the case of farm workers and visitors upon entrance and exit. The study highlighted the link between biosecurity and animal welfare in turkey production, concluding that enhanced biosecurity measures correlate with better welfare outcomes and emphasizing the need for comprehensive and well-implemented biosecurity protocols.

## Introduction

Turkey meat provides high-quality lean protein with essential amino acids and low saturated fat levels, with clear health benefits for consumers (Al-Baidhani and Al-Qutaifi, 2021; Jilo and Hasan, 2022). Additionally, turkey meat production is economical due to the efficient feed conversion rate and low production costs (Kálmán and Szőllősi, 2023). Because of the above, turkey meat has become popular for maintaining a healthy weight and reducing the risk of chronic diseases such as heart problems, obesity, and diabetes (Al-Baidhani and Al-Qutaifi, 2021). Turkey breeders seek options to increase productivity and fulfill growing demands. However, selective breeding for traits like rapid growth and large muscles has led to significant welfare problems for turkeys, including skeletal and muscular issues and painful leg disorders (Erasmus, 2018; Hafez and Shehata, 2021). Moreover, commercially raised turkeys often live in overcrowded conditions, restricting natural behaviors (Kovács et al., 2020). Catching, transporting, and slaughtering them also can induce stress and suffering (Erasmus, 2018). The growing consumer consciousness about the origin of feedstuffs emphasizes the importance of biosecurity practices and animal welfare assessments in ensuring the ethical and safe production of meat (Scott et al., 2018; Alarcón et al., 2021).

Biosecurity, initially linked to biological weapons and bioterrorism, has expanded to various sectors such as agriculture, laboratory safety, and public health. The Food and Agricultural Organization (FAO) defines biosecurity as a strategic and integrated approach encompassing policies and regulatory frameworks to manage risks to human, animal, and plant health and associated environmental risks. It also includes food safety, management of animal and plant diseases, use of living-modified organisms (LMOs) and genetically modified organisms (GMOs), and invasive species management (FAO, 2007). Biosecurity is divided into external and internal main components (Dewul and van Immerseel, 2019). In general, external biosecurity involves measures to prevent the introduction of disease-causing agents from outside the farm. Practices include controlling visitor and personnel movement, using personal protective equipment and decontamination procedures, limiting traffic, regulating animal and product imports, disinfecting incoming materials, and preventing contamination through feed, water, vermin, or birds (Owen, 2017; Huber et al., 2022). Internal biosecurity measures aim to minimize the spread of diseases within a controlled environment. Key practices include cleaning and disinfecting facilities, barn-specific personal protective equipment (PPE), managing stocking densities, following all-in-all-out procedures, and separating susceptible or diseased individuals (Owen, 2017; Dewul and van Immerseel, 2019). Biosecurity is crucial in reducing the use of antimicrobial drugs and minimizing the need for veterinary interventions (Mallioris et al., 2023). A study on global antibiotic consumption from 2000-2010 showed a significant increase in antibiotic use, highlighting the importance of biosecurity in managing antibiotic resistance (Van Boeckel et al., 2014). Implementing biosecurity measures has economic benefits, as they are associated with better economic outcomes in dairy (Osawe et al., 2022) or poultry farms (Siekkinen et al., 2012). In poultry production, the focus has shifted towards prevention, with biosecurity being a key pillar alongside vaccination programs and good management practices (Butcher and Miles, 2019). Effective biosecurity practices create an environment where diseases are less likely to spread and where livestock can thrive (Yadav and Jha, 2019; Butucel et al., 2022). Biosecurity measures, therefore, are supposed to help maintain livestock health and welfare. The predecessor of the Biocheck.Ugent protocol used in our study was first used at Ghent University to survey pig farms (Ribbens et al., 2008). The questionnaire was subsequently developed for several animal species (Dewulf et al., 2019), the results of which can be processed online (https://biocheckgent.com/en).

Animal welfare is just as crucial as biosecurity in production efficiency. Like in other livestock production sectors, the welfare of turkeys throughout their growth and development is essential for producing quality meat while upholding ethically sustainable practices. Animal welfare is defined as the state of an animal as it copes with its environment, with failures or difficulties in coping indicating poor welfare (Broom, 1986, 1991). Suffering, involving negative emotions like fear and pain, is an essential aspect of welfare (Dawkins, 2008). When evaluating welfare using animal-based indicators, modern assessments consider physical and mental states (Webster, 2005, 2022). Assessing turkey welfare involves animal-based indicators, as the Animal Welfare Indicators (AWIN) protocol recommends, along with management and resource-based indicators. This approach provides a comprehensive view of welfare at the farm level, covering aspects like immobility, size, wound types, and some management strategies (AWIN, 2015). Animal-based indicators are considered most relevant and appropriate for evaluating animal welfare (EFSA, 2012).

In our opinion, biosecurity and animal welfare are two sides of the same coin, namely, animal hygiene. Appropriate animal husbandry technology and on-farm management are necessary to maintain animal welfare at an appropriate level. Animals in inadequate housing environments (stress factors) are more susceptible to infection by infectious diseases (Hoffmann et al., 2020). Reducing stress and providing freedom to express natural behavior can positively impact the final profitability of the production (Vigors et al., 2021).

In light of the above-mentioned meaningful connection between biosecurity and animal welfare, this research aimed to quantify the correlation between biosecurity and animal welfare measures in turkey farming by quantifying observable indicators using the Biocheck.UGent questionnaire and the AWIN welfare assessment protocol for turkeys.

## Materials and methods

### Farms and animals investigated

**Fig. 1:**
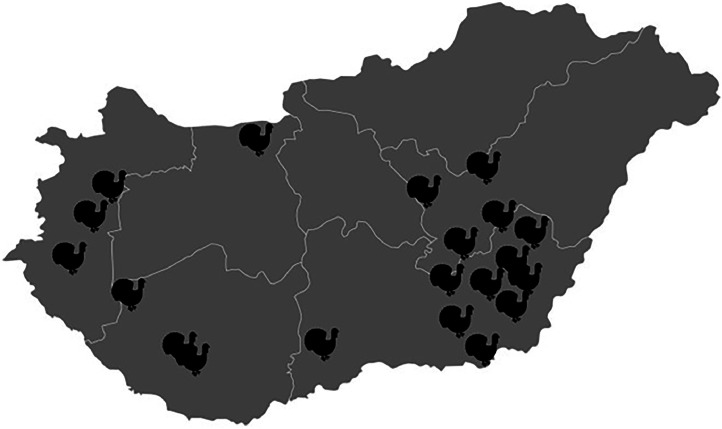
Location of the studied farms.

**Fig. 2:**
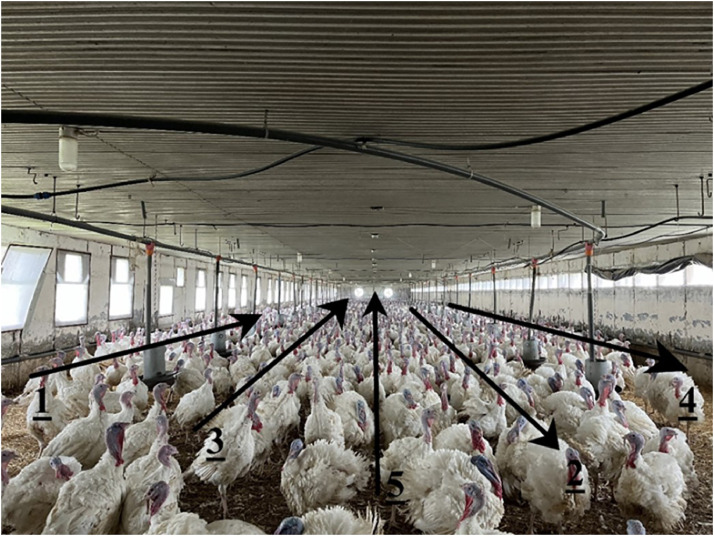
The structure of the transect walks in the assessed flocks. The numbers refer to the order of transect walks. During the walk, the flock inspection should be carried out in the barn in ascending order of figure numbers.

Farms raising male fattening turkeys across Hungary were contacted, with 24 agreeing to participate (Fig. 1). Data protection regulations ensure that the name and location of farms are not published. The inspections focused on male turkey flocks nearing slaughter, averaging 132 days old (range: 98-145 days). Assessments were conducted in half of the turkey barns randomly chosen, averaging 3.7 out of 8.6 buildings per farm. The selected barns had an average floor space of 972 m² (min.: 360; max.: 2000). Farm sizes ranged from 3,000 to over 40,000 turkeys, raised in intensive enclosed buildings with windows on the side (Fig. 2). The turkeys had ad libitum access to feeders and drinkers. Some data from the farms are displayed in Table 1.

**Table 1:** Data of the farms involved in the study.

Farm	No of barns	Farm capacity (birds)	Density (bird/m^2^)	No. of birds at the time of the visit	Age of birds at the time of the visit (days)	Biosecurity score (%)	External biosec. (%)	Internal biosec. (%)	Welfare index
1	8	29000	4.0	7 250	134	77	74	83	4.5
2	6	23760	4.0	7 920	145	68	66	73	5.9
3	14	51100	3.7	7 300	120	66	64	70	8.5
4	6	31800	3.5	14 914	98	62	58	70	2.6
5	15	57000	4.4	26 819	119	54	51	62	5.0
6	12	30000	2.4	13 183	145	74	72	79	3.7
7	10	39500	4.0	18 802	139	64	62	70	2.1
8	6	27960	4.7	13 393	137	65	63	70	3.0
9	11	29700	3.2	13 825	142	39	36	47	14.0
10	6	21960	3.6	10 343	125	51	51	52	9.4
11	6	25980	4.4	12 353	131	52	52	51	4.3
12	7	18970	3.3	8 812	128	40	38	44	9.7
13	15	18990	3.5	8 897	128	40	36	50	6.0
14	6	21000	3.6	9 881	132	43	41	49	6.8
15	10	21000	5.2	9 923	138	46	45	48	5.9
16	10	53000	5.0	13 400	140	51	52	47	5.9
17	8	40000	4.8	10 600	140	51	52	48	7.6
18	10	21000	5.1	9 713	140	41	39	46	7.4
19	10	55000	5.8	25 080	135	48	47	50	8.2
20	4	28000	4.0	13 286	126	46	46	46	7.8
21	8	23000	3.1	7 608	131	61	60	63	6.3
22	3	10000	3.7	4 725	127	46	47	42	11.0
23	3	10000	4.5	4 705	137	45	46	42	14.2
24	1	6000	3.0	2 883	131	49	47	54	5.9

### The biosecurity protocol

The Biocheck.UGent questionnaire for turkey flocks (Biocheck.UGent, 2024; Amalraj et al., 2024; https://biocheckgent.com/en/questionnaires/turkeys) was used to measure farm biosecurity. It generates scores reflecting biosecurity status through multiple-choice questions. The questionnaire covers basic farm characteristics, seven external biosecurity measures subdivisions, and three internal biosecurity subdivisions. External biosecurity subdivisions assess the biosecurity aspects of infrastructure, organization of farm management, access control, poult purchase, depopulation of barns, feeding and water supply, and waste management. Internal biosecurity subdivisions include questions about disease management, measures between compartments, and cleaning protocols (Table 2). The questionnaire was completed together with the farm representatives during the visits. The answers to the questionnaires were then submitted to the Biocheck.UGent online software (https://biocheckgent.com/en/questionnaires/turkeys/start-questionnaire?language=en) which evaluates the farm's biosecurity status ranging from 0 (no measures implemented) to 100 (all necessary biosecurity measures are implemented) (Gelaude et al., 2014; Van Limbergen et al., 2018; Tanquilut et al., 2020; Amalraj et al., 2024). A higher biosecurity score means a higher biosecurity level on the farm.

**Table 2:** The biosecurity measures and their definitions, according to the Biocheck.UGent assessment protocol (Biocheck.UGent, 2024).

Name of the measures	Definition	Number of questions
External biosecurity	It consists of all the farm's preventive measures to prevent pathogens from entering or leaving the farm.	
Infrastructure	Infrastructure, location, and housing are all the structural elements (tools) necessary for biosecurity housing and farm management operations.	16
Organization	Organization of the farm and supply of materials essential for farm management.	10
Visitors and workers	Control and hygienic measures implemented in case of farm workers and visitors upon entrance and exit.	12
Purchase	Protocol of poult purchase and population of barns with livestock.	7
Depopulation	Protocol of depopulation of barns (e.g. transport of adult animals to the slaughterhouse or other poultry holding premises.)	7
Feed and water	Feed and water provision in the barns.	9
Removal	Manure and carcass removal from the barns of the farm.	13
Internal biosecurity	It encompasses all measures implemented to prevent the spread of pathogens within a farm.	
Disease management	Measures of prevention and control of diseases on the farm.	10
Compartments	Measures implemented between each farm structure.	11
Cleaning	Cleaning and disinfection measures on the farm.	11

### The animal welfare assessment protocol

The AWIN protocol for turkey evaluates welfare conditions using animal-based indicators (AWIN, 2015) and was used to assess the animal welfare status of the farms. The AWIN protocol defines four animal welfare principles: good housing, good feeding, good health, and appropriate behavior. Within these principles, twelve distinct but complementary animal welfare criteria are highlighted (AWIN, 2015). The assessments were conducted by two trained individuals (the first and the second author) who filled out the questionnaire with the farm manager and performed transect walks in randomly selected barns on each farm. Their assessment scores showed good agreement during the training. The assessed barns' width was divided into 2-3m wide transects, with an average of five transect walks per building (Fig. 2.; Marchewka et al., 2015; Hrženjak et al., 2021). Turkeys were assessed for head, back, and tail wounds, immobility, lameness, size, feather loss, dirtiness, sickness and death, and behavior according to the AWIN protocol (Table 2). The animals showing signs of the mentioned indices were counted, and their numbers were expressed as a percentage of the total number of animals in the barn. We calculated a total welfare index for each farm by aggregating the welfare indices with the modification of the method by BenSassi et al. (2019) and Averós et al. (2022). Those authors added up the % of the welfare indicators that corresponded to welfare issues of the same nature. We kept the AWIN protocol's weighting, as some indices belong to more than one welfare principle or criteria. The percentage of lame animals, for instance, is counted both in the 'Absence of disease' and the 'Absence of pain', or the percentage of featherless animals is counted in 'Thermal comfort' and also in 'Expression of social behavior' criteria (Table 3). Each indicator was included in the aggregated score as often as in the AWIN protocol to generate objective descriptive outputs. Since the welfare index is a sum of the percentage number of measured items, the lower scores stand for the better welfare situation on the farm.

**Table 3:** The welfare indices measured during the transect walks (AWIN, 2015).

Welfare principle	Welfare criteria	Indicator assessed	Visual cue
Good health	Absence of disease	Immobility	Not moving when approached or encouraged to move
		Lameness	Moving away from the assessor and stopping to rest after a few steps
		Sick	Clear signs of impaired health, resting position, missing body parts
		Terminally ill	Immobile on the ground, feeble breathing, half-closed eyes
		Dead	No breathing and immobile
	Absence of injury; Expression of social behavior	Head wounds	Injury to the head, beak, snood and neck
		Back wounds	Injury to the area between the neck and tail onset
		Tail wounds	Injury to the tail area
Good housing	Thermal comfort; Expression of social behavior	Featherless	Missing feathers on extended areas of the body
	Comfort around resting	Dirtiness	Dark staining of plumage on at least 50% of the body area
Good feeding	Absence of prolonged hunger Absence of prolonged hunger thirst; Absence of disease;	Small size	Visibly smaller size than flock average
Appropriate behavior	Expression of social behavior	Aggression towards mate	Chasing, pecking, flying and flapping aggressively
		Mating	Attempts to sit on each other

### Statistical analysis

Welfare indices, external, internal, and total biosecurity scores were calculated and recorded for each farm. Subscores for each biosecurity measure (within external and internal biosecurity, respectively) were also recorded. The relationship between the welfare index and total, external, and internal subtotal biosecurity scores and categorical subscores was studied by graphical exploration of the data. Having observed linearity, 'Pearson's product-moment correlation was chosen as a proper measure of association between the studied parameters. Pearson's correlation coefficients between 1 (strong positive association) and -1 (strong negative association). In conditions of applicability and the interpretation of the correlation coefficient, the review of Schober et al. (2018) was followed. A p-value below 0.05 was set as a significance level for the obtained correlation coefficients. Correlation coefficients inform about the weak, moderate, or strong association between the welfare index and the other numerical measures (Schober et al.; 2018) but do not say much about the predictive value of these numerical measures. Moreover, pairwise correlation coefficients between the welfare index and individual biosecurity measures do not provide information about the possible collinearity between them. To overcome these statistical issues and to be able to understand better the influence each biosecurity has on the final welfare index of a given farm, the random forest classification and regression algorithm were used. The random forest algorithm works by fitting numerous decision trees to a subset of the data (training set) and tests the predictive efficiency of decision trees on the remaining part of the dataset (test set). A decision tree is a flowchart-like model used in machine learning to make decisions based on input features. It looks for associations between the predictor variables and the outcome by identifying cutoff values in the predictor variables that divide the dataset into more or less homogenous subsets in terms of the outcome variable and, therefore, could serve as a basis for classification or numerical prediction. Each branch represents a yes/no decision (based on a given cutoff value of the predictor variable), while leaves (endpoints) represent the prediction of the outcome. The random forest algorithm synthesizes the information of several decision trees, each working with only a subset of predictor variables, randomly chosen from all predictor variables. This algorithm can handle the problem of small sample size beside many parameters, collinearity, and interactions between predictor variables (biosecurity scores and subscores) when identifying those that matter most in predicting the outcome parameter (the welfare index). The ‘importance’ of predictor variables, that is, which has the biggest influence on the outcome parameter, can be determined by the permutation method proposed by Breiman (2001). This method compares the predictive accuracy of the test set before and after permuting the values of one of the outcome variables. The prediction accuracy is expressed as a loss function, usually the mean square of differences between the predicted and observed value of the outcome parameter. If the prediction accuracy drops, the given predictor variable plays a major role in predicting the outcome variable. By sorting the differences between before and after permutation accuracy, the predictor variables can be ranked in terms of their influence or importance. We used a visual representation to display the order variables. All statistical analysis was performed with the *base, corrupt*, and *party* packages of the statistical software R (R Core Team, 2024).

## Results

**Fig. 3:**
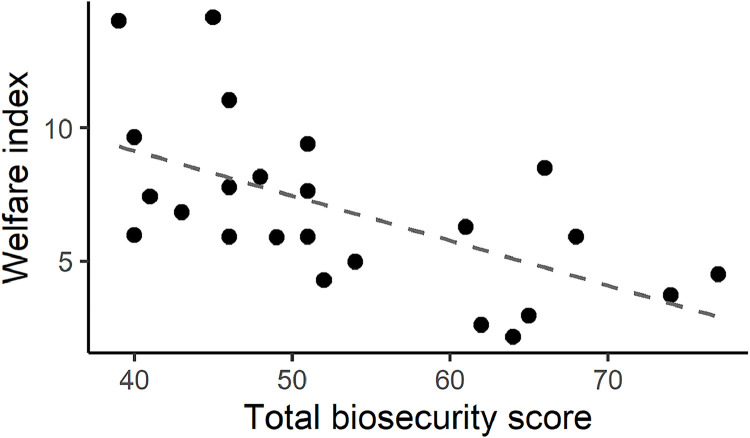
Point estimates of the correlation between the welfare index and total biosecurity score of the farms enrolled. The least-squares line is displayed for descriptive purposes only.

**Fig. 4:**
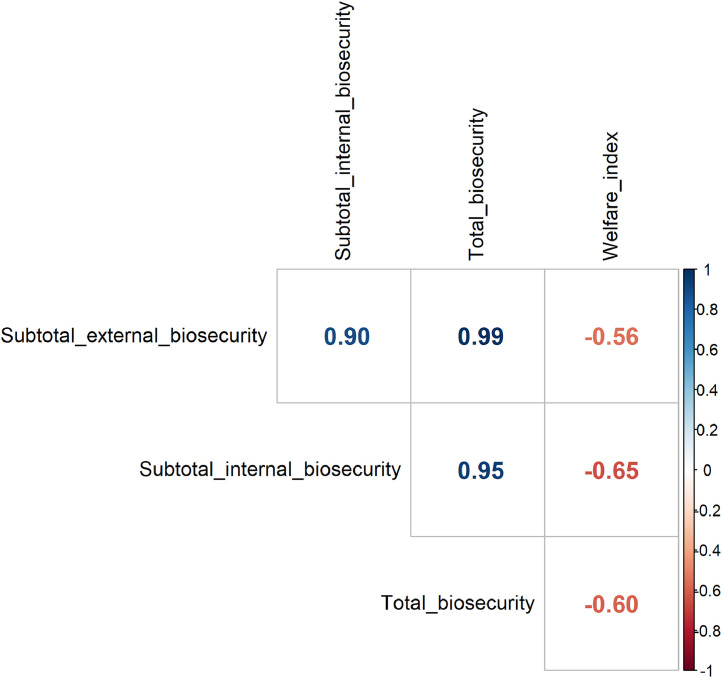
Point estimates of correlation coefficients between the welfare index and the biosecurity subfolders.

The average welfare index was 6.9 (min.: 2.1; max.: 14.2) on the farms enrolled, while the average total, external and internal biosecurity scores were 53.3 (min.: 39; max.: 77), 51.9 (min.: 36; max.: 74), 56.5 (min.: 42; max.: 83), respectively. The explorative analysis revealed a pattern of inverse proportionality between the welfare index and total biosecurity scores (Fig. 3). It was further explored by correlation testing, including the correlation of the welfare index with external and internal biosecurity subtotals, too (Fig. 4). Besides the expected strong correlation between total biosecurity scores and subtotals (0.95-0.99), there was a significant moderate negative correlation between the welfare index and the total biosecurity score of the farms (r = -0.597 (95 %CI: -0.256; -0.806; p = 0.0021)).

**Fig. 5:**
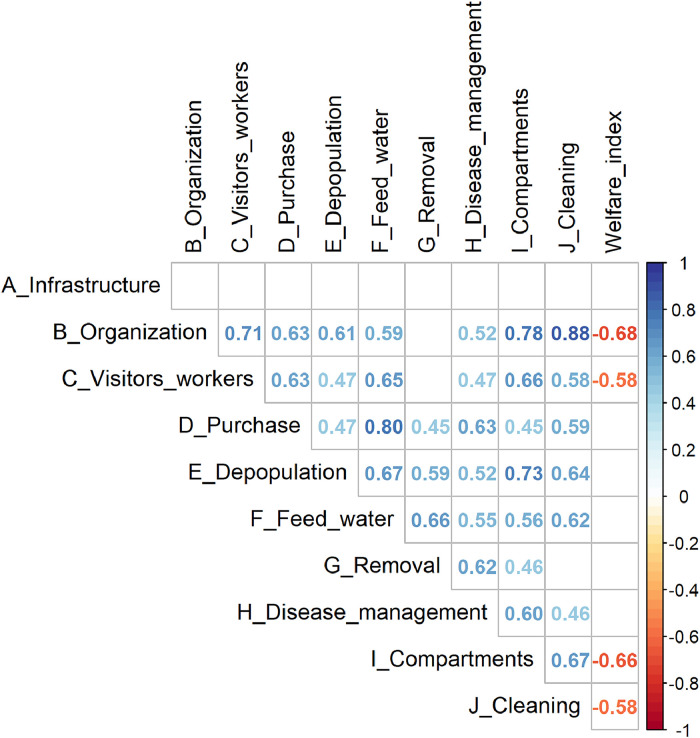
Point estimates of correlation coefficients of statistically significant Pearson's product-moment correlation between the welfare index and the biosecurity category subscores.

Fig. 5 shows the correlation between the subscores for biosecurity with each other and the welfare index, separately. Following the thresholds proposed by Schober et al. (2018) for interpreting the magnitude of the correlation coefficient, a statistically significant correlation of moderate strength (between 0.4 – 0.69) was found between the welfare index and the subscores for the 'organization' of the farm, the hygiene measures for 'visitors and workers' and the measures between 'compartments' and subscore for 'cleaning', respectively.

**Fig. 6:**
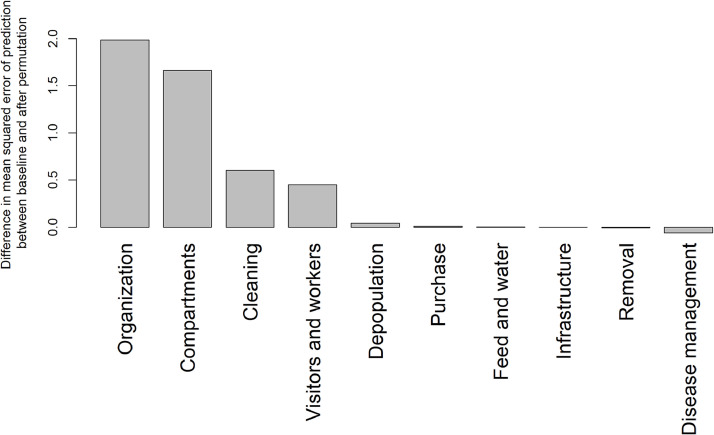
Ranking of variable importance in the prediction of welfare index based on the random variable forest algorithm. The height of the bars is proportional to the increase in the error of the prediction of the welfare index (value of the loss function) due to the permutation of the values of the given predictor variable.

Fig. 6 shows the order of variable importance based on the results of the permutation method used in the random forest algorithm. Based on the random forest algorithm, the score for how organized farm management is, the safety level of hygiene measures between compartments, cleaning and disinfection measures on the farm, and hygiene measures implemented regarding visitors and farm workers carry the most information regarding the prediction of the magnitude of the welfare index.

## Discussion

Our study revealed a significant correlation between biosecurity and animal welfare on turkey farms, suggesting that higher biosecurity levels are associated with better animal welfare outcomes. This finding aligns with previous research that emphasizes the role of robust biosecurity measures in enhancing animal health and welfare (Scott et al., 2018; Siekkinen et al., 2012). Specifically, biosecurity practices such as stringent disinfection protocols, controlled access to poultry houses, and effective disease monitoring systems can mitigate the risk of infections, thereby improving the overall health and welfare of the flock (Dewul and van Immerseel, 2019).

The separate investigation of biosecurity into external and internal measures provided additional insights. External biosecurity, which includes measures to prevent the introduction of pathogens onto the farm, showed a significant correlation with welfare indices. This finding is consistent with the literature, where external biosecurity is often highlighted as critical for preventing the entry of infectious agents (Guinat et al., 2020; Hald et al., 2000). Effective external biosecurity measures, such as quarantine protocols for new birds and restricting visitor access, are crucial in maintaining a pathogen-free environment, directly impacting animal welfare.

Internal biosecurity, focusing on practices within the farm to control the spread of pathogens, also correlated significantly with welfare outcomes. Regular cleaning and disinfection measures, proper waste management, and good ventilation within poultry houses are essential in controlling disease spread and reducing stress (Tanquilut et al., 2020). The significance of both external and internal biosecurity highlights the need for comprehensive plans that address all potential pathways of pathogen transmission (Butucel et al., 2022). There is a strong correlation between external and internal biosecurity scores, as displayed in Fig. 4, being in line with others' results (Caekebeke et al., 2020; Tilli et al., 2022; Mallioris et al., 2023).

The random forest algorithm identified several key biosecurity practices significantly influencing welfare indices. 'Organization', 'compartment', 'cleaning', and 'visitors and workers' were the most decisive variables. 'Organization' and 'visitors and workers' are key elements of external biosecurity, while 'compartment' and 'cleaning' are essential principal parts of internal biosecurity (Dewul and van Immerseel, 2019). This also underlines the importance of organized and structured biosecurity protocols. An integral part of the organization is, among other things, a written biosecurity plan with disease prevention rules to prevent the introduction and spread of pathogens within the poultry farm (Tilli et al., 2022; Delpont et al., 2023). For instance, well-defined compartments and controlled movement between them can prevent cross-contamination within the farm (Chantziaras et al., 2014). Regular and thorough cleaning practices are fundamental in reducing pathogen load in the environment, directly impacting the health and welfare of the birds (Ssematimba et al., 2013). An important method of developing a biosecurity plan includes looking at the conceptual, structural, and operational components of an integrated poultry operation (Martin, 2017).

A study assessed the biosecurity compliance in 259 poultry farms in Northeast Italy, showing a generally high level of biosecurity, particularly in turkey farms. However, some deficiencies were noted, such as cleanliness issues in broiler farms and inadequate coverage of built-up litter in turkey and broiler farms (Tilli et al., 2022). These findings highlight the importance of strict national regulations and the integrated nature of the poultry industry in ensuring high biosecurity standards. Operational factors, such as employee training and proximity to residential areas, also contribute to biosecurity, underscoring the necessity of education and structural improvements to enhance biosecurity practices in diverse geographical settings (Tsegaye et al., 2023). Farms implementing comprehensive biosecurity and welfare measures had healthier flocks with lower mortality rates, suggesting that welfare considerations should be essential to biosecurity planning. Integrated approaches addressing biosecurity and welfare led to the best health outcomes in the study of Butucel et al. (2022). Another study assessed the level of biosecurity and welfare in farm animals, finding that systems with higher welfare standards tended to have better biosecurity practices (da Costa and Diana, 2022). This positive relationship indicates that improving welfare can also enhance biosecurity outcomes, creating a beneficial cycle for animal health and farm productivity. This reinforces the need for a holistic farm management approach that prioritizes both health and welfare to achieve optimal results.

The significant correlations between biosecurity scores and welfare indices in our study suggest that improving biosecurity can lead to better welfare outcomes. This is particularly important for large-scale turkey farms, where high bird densities can exacerbate the spread of diseases and welfare issues (Erasmus, 2018; Kovács et al., 2020; Hafez and Shehata, 2021). Implementing rigorous biosecurity measures can reduce disease incidence, lower the need for antimicrobials, and enhance animal welfare (Dhaka et al., 2023). Of course, correlation does not necessarily mean causation. Other factors besides biosecurity may affect animal welfare, such as human-animal relationships (Mota-Rojas et al., 2020). The AWIN protocol for turkeys does not include the assessment of human-animal relationships, therefore, we can not evaluate the effects of the workers' behavior on animal welfare.

Our findings support the importance of integrating biosecurity and welfare assessments in routine farm management practices. Tools like the Biocheck.Ugent questionnaire and the AWIN protocol provide valuable frameworks for evaluating and improving both biosecurity and welfare standards (Gelaude et al., 2014; AWIN, 2015). By using these tools, farmers can identify areas of improvement and implement targeted measures to enhance biosecurity and welfare. Key challenges and improvement areas, such as inconsistent data collection and lack of standardized practices, can also be overcome through better data sharing and standardized assessment methods (Delpont et al., 2023).

## Conclusions

This study highlights the link between biosecurity and animal welfare in turkey production. Enhanced biosecurity measures correlate with better welfare outcomes, emphasizing the need for comprehensive and well-implemented biosecurity protocols. Future research should continue to explore these relationships, focusing on the long-term impacts of biosecurity improvements on animal welfare and productivity. Adopting a holistic approach that integrates biosecurity and welfare considerations can lead to sustainable and ethically responsible poultry production systems.

## Declaration of competing interest

The authors declare that they have no known competing financial interests or personal relationships that could have appeared to influence the work reported in this paper.
